# Secondary severe thrombocytosis in a patient who underwent splenectomy due to hereditary spherocytosis and its treatment using hydroxyurea

**DOI:** 10.11604/pamj.2019.32.175.17841

**Published:** 2019-04-10

**Authors:** Hakan Sarbay, Sinan Akbayram

**Affiliations:** 1 Diyarbakir Children Hospital, Pediatric Hematology and Oncology, Turkey; 2 Gaziantep University Faculty of Medicine, Pediatric Hematology and Oncology, Turkey

**Keywords:** Hydroxyurea, thrombocytosis, splenectomy

## Abstract

Thrombocytosis is a frequently seen condition during childhood. While it usually develops secondarily due to reasons such as infection or anemia, it may rarely develop due to clonal causes. Thrombocytosis becomes a life-threatening condition by causing severe complications such as hemorrhage and thrombosis development. Treatment is not recommended in patients who are asymptomatic and with a platelet count below 1,500,000/mm^3^, however, treatment is required in cases who are symptomatic and with a platelet count above 1,500,000/mm^3^ in conditions such as primary thrombocytosis. This article present the outcomes of a patient who was treated using low-dose hydroxyurea when he developed severe thrombocytosis after splenectomy.

## Introduction

Thrombocytosis is one of the common hematological findings seen during childhood. The most common causes include infections, anemia, autoimmune diseases and splenectomy [1]. Rarely, thrombocytosis may also develop due to clonal causes. The most prominent examples of this include chronic myeloid leukemia and essential thrombocytosis [2]. The first step to approach thrombocytosis is to repeat the complete blood count test to rule out the false high values caused by cellular destruction. When thrombocytosis is confirmed, it is necessary to classify the thrombocytosis based on its severity. Studies have divided thrombocytosis into four groups based on the risk of complication development: mild thrombocytosis (500,000-700,000/mm^3^), moderate thrombocytosis (700,000-900,000/mm^3^), severe thrombocytosis (>900,000/mm^3^) and very severe thrombocytosis (>1,000,000/mm^3^) [3, 4]. This article present the outcomes of a patient who was treated using low-dose hydroxyurea when he developed severe thrombocytosis after splenectomy.

## Patient and observation

Fifteen-year-old male patient who underwent splenectomy 5 years ago due to hereditary spherocytosis, massive splenomegaly and hypersplenism applied for outpatient clinic visit. Having no complaints other than fatigue and intermittent abdominal pain, the patient was receiving penicillin prophylaxis and acetylsalicylic acid (100mg/day) treatment. Laboratory results included a white blood cell count of 18.760/uL, hemoglobin of 12.7gr/dL, platelet count of 2,235,000/uL, ferritin of 68ng/mL, Vitamin B12 of 262ng/mL and folate of 6.1ng/mL and liver and kidney functions tests were within normal limits. The patient was hospitalized for IV hydration therapy. Acetylsalicylic acid treatment was continued. Peripheral smear leukocyte formula included neutrophil of 58%, lymphocyte of 36%, monocyte of 4% and eosinophil of 2%. No atypical cell or blast was observed. Mild hypochromia, microspherocytes and large clusters of platelets were observed. In the follow-up analyses after hydration therapy, platelet counts were above 2,000,000/uL. Analysis for Janus kinase (JAK) mutation was sent. Hydroxyurea was initiated at a dose of 1x500mg/day (10mg/kg/day). Platelet count regressed to 1.630.000/uL at week 2 and to 1,568,000/uL at week 4. Experiencing no clinical or laboratory side effect in the weekly follow-ups, the patient had a platelet count of 1,440,000/uL at the end of week 6 (Table 1). Hydroxyurea dose was increased to 15mg/kg/day. As the patient developed dyspeptic complaints, the dose of the medication was decreased back to 10mg/kg/day. The patient is still being followed up.

**Table 1:** laboratory values of the patient

	1. Day	1. Week	2. Week	4. Week	5. Week	6. Week
Plt	2,010,000/uL	1,596,000/uL	1,630,000/uL	1,568,000/uL	1,474,000/uL	1,440,000/uL
WBC	12,790/Ul	17,660/uL	18,280/uL	11,780/uL	12,390/uL	12,450/uL
Neut	6,110/Ul	10,570/uL	9,250/uL	5,640/uL	4,890/uL	6,290/uL
Hgb	10.8 g/dl	11.7 g/dl	11.8 g/dl	12.2 g/dl	12.4 g/dl	11.9 g/dl
RBC	5.1x10^6^/uL	5.3x10^6^/uL	5.5x10^6^/uL	5.6x10^6^/uL	5.7x10^6^/uL	5,5x10^6^/uL
Urea	19 mg/dl	13 mg/dl	21 mg/dl	12 mg/dl	8 mg/dl	23 mg/dl
Creat	0.5 mg/dl	0.6 mg/dl	0.6 mg/dl	0.6 mg/dl	0.7 mg/dl	0.5 mg/dl
AST	35 U/L	56 U/L	39 U/L	47 U/L	59 U/L	25 U/L
ALT	16 U/L	31 U/L	22 U/L	31 U/L	30 U/L	28 U/L
Uric asid	3.7 mg/dl	-	4.2 mg/dl	-	4.2 mg/dl	4 mg/dl
Glucose	89 mg/dl	-	-	89 mg/dl	87 mg/dl	96 mg/dl
Adverse Reaction	-	-	-	-	-	-

Plt: Platelet, WBC: White blood cell, Neut: Neutrophil, Hgb: Hemoglobin, RBC: Red blood cell, Creat: Creatinine, AST: Aspartate aminotransferase, ALT: Alanine aminotransferase

## Discussion

Pathophysiologically, thrombocytosis is divided into two groups as primary and secondary. Primary causes include essential thrombocytosis and familial thrombocytosis. As with other myeloproliferative diseases, essential thrombocytosis is more common in adult age groups and rarely seen during childhood. Secondary thrombocytosis is more common during infancy and its incidence decreases with increasing age [5, 6]. While the patient's thrombocytosis has been considered to be due to splenectomy, Janus kinase 2 (JAK2) mutation was studied to rule out essential thrombocytosis. While thrombocytosis is mostly asymptomatic, high platelet count may cause symptoms and signs such as headache called vasomotor phenomenon, visual disturbances, fatigue, chest pain, abdominal pain, vertigo, aphasia and dysarthria independent from the etiological causes [5]. Our case had the complaints of fatigue and intermittent abdominal pain. Platelet count was above 2,000,000/mm^3^ and taking the splenectomy into account, the risk of thrombosis was considered to be high. The most important point in the follow-up and treatment of thrombocytosis is to determine the etiology. In conditions such as infection, anemia and inflammatory diseases, thrombocytosis regresses when the underlying condition is treated [6]. However, thrombocytosis may be more resistant after splenectomy. Therefore, longer treatment may be required compared to the other etiological conditions. The most commonly used medication in treatment is acetylsalicylic acid. Thromboxane causes a decrease in platelet activity by suppressing A2 production [3]. Our patient has been receiving acetylsalicylic acid treatment for almost 5 years. Thrombosis was not observed despite platelet counts were high in follow-up analyses. However, due to high platelet count, hydroxyurea treatment was planned to be initiated. In the literature, cytoreductive therapy has been mostly initiated in essential thrombocytosis and when the platelet count is >1,500,000/mm^3^ [7, 8]. It is recommended to start the treatment at a dose of 15mg/kg/day and adjust the dose based on follow-up. While there is a risk of leukemic transformation at long term, it was found to be well-tolerated, effective in reducing the platelet count and reduce the risk of thrombotic events [9, 10]. In our case, hydroxyurea was initiated at a dose of 10mg/kg/day. During the follow-up, the platelet count regressed below 1,500,000/mm^3^ and no side effect was observed. The dose was increased to 15mg/kg/day. However, as dyspeptic complaints developed after dose increase, the dose was reduced back to 10mg/kg/day.

## Conclusion

There are case reports of successful use of hydroxyurea in patients with essential thrombocytosis in the literature [7, 8]. We emphasize that in patients with secondary thrombocytosis, low-dose hydroxyurea treatment can be used safely when the platelet count is above 1,500,000/mm3 for a long period of time and the risk of thrombosis is high, and platelet count can be reduced to safe levels.

## Competing interests

The authors declare no competing interests.
